# Risk Factors of Ureteral Stenosis After Percutaneous Microwave Ablation of Renal Tumor, a Single-Center Experience

**DOI:** 10.3389/fonc.2020.521349

**Published:** 2020-09-18

**Authors:** Jiapeng Wu, Jie Yu, Zhigang Cheng, Zhiyu Han, Xiaoling Yu, Kai Li, Ping Hu, Fangyi Liu, Ping Liang

**Affiliations:** ^1^Department of Interventional Ultrasound, Chinese PLA General Hospital, Beijing, China; ^2^State Key Laboratory of Kidney Disease, The Chinese PLA General Hospital, Beijing, China; ^3^Ultrasonic Department, The Second Affiliated Hospital of Chengdu Medical College, Nuclear Industry 416 Hospital, Chengdu, China; ^4^Department of Ultrasound, First People’s Hospital of Datong, Datong, China

**Keywords:** microwave ablation, renal tumor, complication, stenosis of the ureter, R.E.N.A.L. score

## Abstract

**Background:**

Ureteral stenosis after percutaneous microwave ablation (MWA) of renal tumor is a rare but severe complication, and its risk factors are not apparent.

**Purpose:**

This study aimed to investigate the risk factors for stenosis of ureter after MWA treatment of the renal tumor that is a rare complication.

**Materials and Methods:**

Data of 211 patients who underwent MWA for the treatment of renal tumor were retrospectively analyzed from September 2006 to August 2019. Demographic characteristics, clinical features, ablation parameters, and outcomes were analyzed to find out the potential risk factors of this complication. *P* < 0.05 is considered significant.

**Results:**

Six of 211 patients developed ureter stenosis, and the rate of this complication is 2.84%. The median time of emergence of hydronephrosis was 226 (range, 3–390) days. Univariate analysis shows the distance between ureter and tumor (*P* = 0.225) or ablation zone (*P* = 0.089) is not related to this complication. Postoperative urine routine (red blood cell, *P* = 0.001; white blood cell, *P* = 0.035) and R.E.N.A.L. score (*P* < 0.001) is related to this complication. But after multivariate logistic analysis, only R.E.N.A.L. score (*P* = 0.004) is associated with this complication. The location and growth pattern of tumor and the energy of ablation were not related to this complication independently.

**Conclusion:**

The stenosis of the ureter after MWA of renal tumor is not associated with the tumor size, location, or the distance between the ureter and tumor and ablation site independently. But R.E.N.A.L. score is associated with ureter stenosis after MWA for the treatment of renal tumor, which combines the information of location, depth, and size of tumor. Preoperative evaluation of the tumor is necessary for avoiding ureter stenosis. Further studies should focus on these risk factors of this complication.

## Introduction

The renal tumor is one of the fatal urological malignancies. The incidence of the renal tumor has been growing. However, the survival rate of the renal tumor has increased (1, 2). Although surgical resection is considered as the principal treatment for renal tumor (3), since Zegel used cryoablation (CA) for renal tumor for the first time (4), ablative technique, as a minimally invasively therapy for the treatment of the renal tumor, has been used in the treatment of renal tumors widely and included in the guidelines for the treatment of renal tumors (5).

Microwave ablation (MWA) for the renal tumor is a minimally invasive therapy, which can be performed under the guidance of ultrasound (US) or computed tomography (CT) during the operation or percutaneously directly. It has been proved in the previous study that MWA can achieve a similar effect and lower rate of complication compared with laparoscopic radical nephrectomy and open radical nephrectomy (6–8). The rate of major complications was 1.8%; the rate of minor complication was 17.5% (9). Injury or stenosis of the ureter away from ablation zone after MWA for the renal tumor is a rare but severe complication, and this complication might induce a decrease in quality of life because they might always suffer double-J stent placement or percutaneous puncture catheter drainage (PPCD) caused by the complication. To the best of our knowledge, there are rarely researches of this complication after MWA for renal tumor. Even there appeared reports about this complication, none of these articles investigate the risk factors of this complication (10–12). Here, we report six cases with injury or stenosis of the ureter after MWA for renal tumor and try to find potential risk factors.

## Materials and Methods

### Patients

This study is approved by institutional review board at Chinese PLA General Hospital. This retrospectively study enrolled the 211 patients in the Interventional Ultrasound Department of Chinese PLA General Hospital from September 2006 to August 2019 who had undergone MWA for the treatment of renal tumor. All the patients had signed the informed consent form. The institutional database was queried to identify incident patients and collect baseline clinical data including age, sex, comorbidity, lesion location, ablation time, ablation power, the maximum diameter of the tumor and ablation zone, preoperative, postoperative and follow-up imaging examination, and laboratory examination. Our study was approved by the institutional review board. The collected criteria were as follows: (1) conformed to the treatment guidelines of NCCN (5), (2) refusal of surgery or inability of operation, and (3) stenosis of the ureter after MWA therapy. R.E.N.A.L. score (13) was used to evaluate the tumor size, location, and depth. Because all of the tumors were less than 4 cm, all the scores of tumor size were 1 point. To evaluate exophytic or endophytic property, tumors that are 50% or more exophytic are assigned 1 point, tumors less than 50% exophytic are assigned 2 points, and those that are entirely endophytic are assigned 3 points. To quantitate the distance between collecting system and tumor, the distances that are more than 7 mm is assigned 1 point; 4 to 7 mm, 2 points; and less than 4 mm, 3 points. To evaluate the location of tumor, tumors that are entirely above the upper polar line or below the lower polar line are assigned 1 point. If the mass crossed the polar line, a score of 2 points is given. A tumor that has greater than 50% of the diameter across either polar line, crossed the renal axial midline, or is fully contained between the polar lines is assigned 3 points.

### Technique and Procedure

All the patients are treated with percutaneous MWA under US guidance by experienced doctors. The microwave unit (KY-2000, Kangyou Medical, Nanjing, China) is capable of producing 100 W of power at 2,450 MHz. An automatic biopsy gun with an 18-gage cutting needle to puncture the biopsy for two to three times to achieve tumor tissue was used, followed by 1% lidocaine local anesthesia (Yiyou, Beijing, China). A protective temperature-measured device was inserted to control the temperature of the surrounding tissue. The antenna was then inserted into the mass and placed at a proper location under US guidance. After antennas were placed, intravenous anesthesia was administered by a combination of propofol (Diprivan; Zeneca Pharmaceuticals, Wilmington, DE, United States), and ketamine (Shuanghe Pharmaceuticals, Beijing, China) via the peripheral vein.

Hydrodissection technique is a protective measure to reduce the heat injury of surrounding tissues, and this method has proven its efficiency to avoid the damage to the intestinal tract and renal sinus around (14–16). For patient 2, because the tumor was entirely in the pelvis, saline was injected into the renal pelvis continuously during the ablation procedure.

### Patient Characteristic and Follow-Up

**FIGURE 1:**
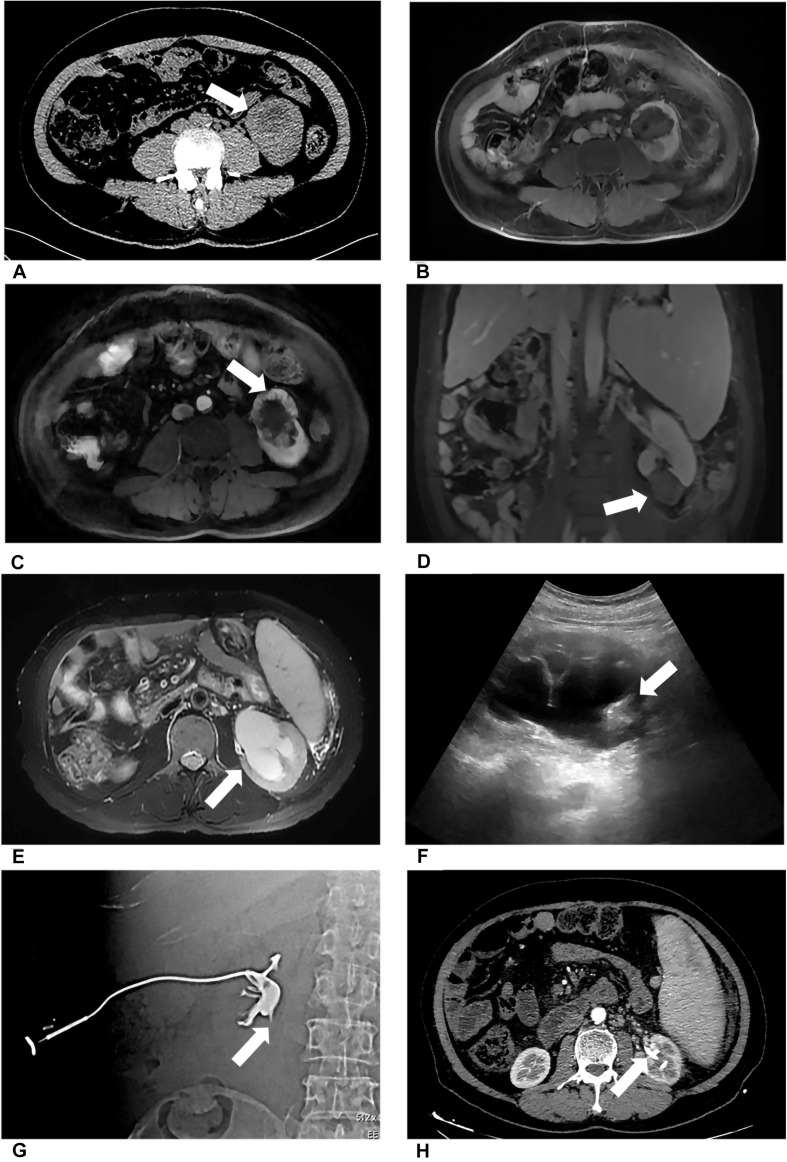
Patient 1 underwent MWA of a 3.6-cm renal tumor in the lower left kidney. 8 months after first MWA, local recurrence was confirmed by MRI, and the patient accepted second MWA. 13 months after second MWA, severe hydronephrosis was detected on his left kidney; the drainage tube was placed in his renal pelvis under US guidance. Digital subtraction angiography (DSA) confirmed serve ureteropelvic junction stricture. **(A)** Axial enhanced CT shows a renal mass (white arrow) in the lower pole of the left kidney. **(B)** Axial MRI shows the ablation site (white arrow) 13 days after MWA. **(C)** Axial MRI shows local recurrence (white arrow) 8 months after the first MWA. **(D)** Coronal MRI scan obtained in the third day after the second ablation (white arrow). **(E)** Axial MRI shows severe hydronephrosis (white arrow) 13 months after the second ablation. **(F)** US image shows the drainage tube (white arrow) after percutaneous puncture catheter drainage under US guidance. **(G)** DSA shows the contrast agent cannot flow through the ureter in the prone position, considering ureteropelvic junction stricture (white arrow). **(H)** Axial CT shows the drainage tube (white arrow) in the renal pelvis and without hydronephrosis 3 months after percutaneous puncture catheter drainage.

Preoperative imaging examination, such as US, contrast-enhanced US, and contrast-enhanced CT/magnetic resonance imaging (MRI), was retrospectively analyzed to determine the tumor location, the diameter of the tumor, and the relationship between the lesion and adjacent structure. The distance between the ureter and tumor or ablation site was measured on MRI/CT. Postoperative first urine routine was collected to analyze potential risk factors. Postoperative imaging examination was retrieved to measure the diameter of the ablation zone and judge if there was stenosis of ureter and secondary hydronephrosis and record the occurrence time of complication. For patient 2, hydronephrosis was detected by US 180 days after ablation, and she accepted percutaneous nephrostomy (Figure 1). Patient 6 complained of abdominal pain 3 days after ablation; CT showed ureterectasia of the upper ureter and hydronephrosis, considering ureter stenosis because of inflammatory edema. After double-J stent placement, abdominal pain achieved relief.

### Statistical Analysis

The data were expressed as median or mean ± SD. The correlation between this complication and clinical characteristics was analyzed using a χ^2^ test or Fisher exact test for categorical variables and Mann–Whitney *U* test for continuous variables. Variables in which *P* value is less than 0.2 or clinically considered meaningful were included into multivariable logistic regression. The statistical analysis was calculated by SPSS 18.0 software package (Chicago, IL, United States) and *R* (version 3.6.1). *P* < 0.05 was considered significant. The receiver operating characteristic curve was plotted by *R* (version 3.6.1).

## Results

### Complication

Six of 211 patients developed ureter stenosis, and the rate of this complication is 2.84%. The characteristic baseline is given in Table 1, and complication-related information of these six patients and tumors is given in Table 2. Of these six patients, the mean distance between tumor and ureter is 22.2 mm (range, 12.0–35.0 mm). The mean distance between ablation site and ureter is 20.1 mm (range, 10.6–32.1 mm). Four of six patients developed hydronephrosis. The median time of emergence of hydronephrosis was 226 (range, 3–390) days. After ablation, two patients showed macroscopic hematuria. Minor and major complication is shown in Supplementary Table 1. The patients who had severe hydronephrosis and accepted double-J stent placement or PPCD and accepted medical image examination every 3 months. After double-J stent or drainage catheter placement, the hydronephrosis was relieved. During the follow-up time, the drainage might cause blockage or exodus. And catheter placement again under US guidance was considered.

**TABLE 1:** Characteristic of the patients at baseline.

Characteristic	*n* = 211
Median age (range; years)	63.3 (21–90 years)
Sex, no. (%)	
Male	151 (71.6%)
Female	60 (28.4%)
Tumor diameter (cm)	2.71 ± 0.73
**Preoperative urine routine**	
RBC (μL)	2.76 ± 7.32
WBC (μL)	17.4 ± 80.2
Urine protein (mg/dL)	17.3 ± 59.0
The distance between ureter and tumor (mm)	24.3 ± 12.7
The distance between tumor and sinus (mm)	6.71 ± 5.5
**RENAL score** 4 5 6 7 8 9 10	12 (5.7%) 53 (25.1%) 46 (23.2%) 34 (16.1%) 43 (23.2%) 16 (7.6%) 5 (2.4%)

*RBC, red blood cell; WBC, white blood cell.*

**TABLE 2:** Characteristic of patients with ureter stenosis.

Patient no.	1	2	3	4	5	6
Age	85	78	82	57	79	68
Sex	Male	Female	Male	Male	Male	Male
The distance between ureter and tumor (mm)	12.7	14.5	35.0	12.0	26.0	29.2
The distance between ureter and ablation zone (mm)	11.2	11.9	32.1	10.6	31.2	26.9
The maximum diameter of the tumor (cm)	3.6	3.2	3.3	2.6	3.8	2.5
The maximum diameter of the ablation zone (cm)	3.7	4.7	4.7	4.1	3.9	2.7
Pathologic diagnosis	Clear cell carcinoma	Papillary carcinoma	Clear cell carcinoma	Clear cell carcinoma	Clear cell carcinoma	Clear cell carcinoma
Location	Lower segment	Middle segment	Lower segment	Upper segment	Upper segment	Middle segment
Adjacent to renal pelvis (+/−)	+	+	+	+	+	+
Ablation energy (J)	42,000	42,000	60,000	24,000	60,000	30,000
Macroscopic hematuria (+/−)	−	+	−	−	+	−
Time of emerging hydronephrosis (d)	N/A	330	180	390	N/A	3
Treatment measure	Double-J stent	PPCD, Double-J stent	PPCD	N/A	N/A	Double-J stent
Postoperative urine routine						
Urine erythrocyte (μL)	108.8	3,051.6	18.9	103.5	8,379.0	95.7
Urine leukocyte (μL)	4	662.2	10.8	15	47.7	4.9
Urine protein (mg/dL)	20	70	25	70	100	20
Renal score	9	10	8	10	9	9

*PPCD, percutaneous puncture catheter drainage.*

### Risk Factors of Ureter Stricture

#### Univariate Analysis of Risk Factors

Table 3 shows univariate and multivariate analysis of risk factors for ureter stricture. Among 211 patients, tumor diameter and diameter of ablation (Mann–Whitney *U* test *P* = 0.093, 0.099), ablation power and time (Mann–Whitney *U* test *P* = 0.426, 0.396), total energy (*P* = 0.739), and postoperative urine white blood cell (WBC; *P* = 0.255) were unrelated to this complication. The distance between ureter and tumor or ablation zone (Mann–Whitney *U* test *P* = 0.225, 0.089) was unrelated to this complication. Increased postoperative urine routine [Mann–Whitney *U* test, red blood cell (RBC), *P* = 0.001; urine protein, *P* = 0.035] and R.E.N.A.L. score (Mann–Whitney *U* test *P* < 0.001) were related to this complication.

**TABLE 3:** Univariate and multivariate analysis of risk factors for ureter stricture.

Risk factors	Complication *n* = 6	Without complication *n* = 205	Univariate	Multivariate
			
			*P*	*P*
Tumor diameter (cm)	3.2 ± 0.5	2.7 ± 0.7	0.093	0.099
Diameter of ablation zone (cm)	3.9 ± 0.8	3.4 ± 1.0	0.123	0.765
**Postoperative urine routine**				
RBC (μL)	1,968.6 ± 3,356.0	338.4 ± 2,479.7	0.001	0.125
WBC (μL)	123.3 ± 264.6	19.8 ± 64.6	0.255	0.831
Urine protein (mg/dL)	50.8 ± 33.8	32.4 ± 176.0	0.035	0.224
Ablation power (W)	50 ± 0	50 ± 22.1	0.426	
Ablation time (s)	380 ± 129.6	439.8 ± 176.0	0.396	
Total energy (J)	43,000.0 ± 14,900.0	43,674.0 ± 22,970.0	0.739	0.121
Diabetes	1/5	40/165	0.999	
High blood pressure	1/5	89/116	0.261	
Charlson Comorbidity Index	3.6 ± 2.6	3. ± 1.0	0.535	
The distance between ureter and tumor (mm)	22.0 ± 10.2	24.5 ± 8.4	0.225	0.054
The distance between ureter and ablation zone (mm)	11.6 ± 11.0	20.9 ± 10.0	0.089	0.149
Renal score 4 5 6 7 8 9 10	0 0 0 0 1 3 2	12 53 46 34 42 13 3	<0.001	0.004

*RBC, red blood cell; WBC, white blood cell.*

#### Multivariate Analysis of Risk Factors

**FIGURE 2:**
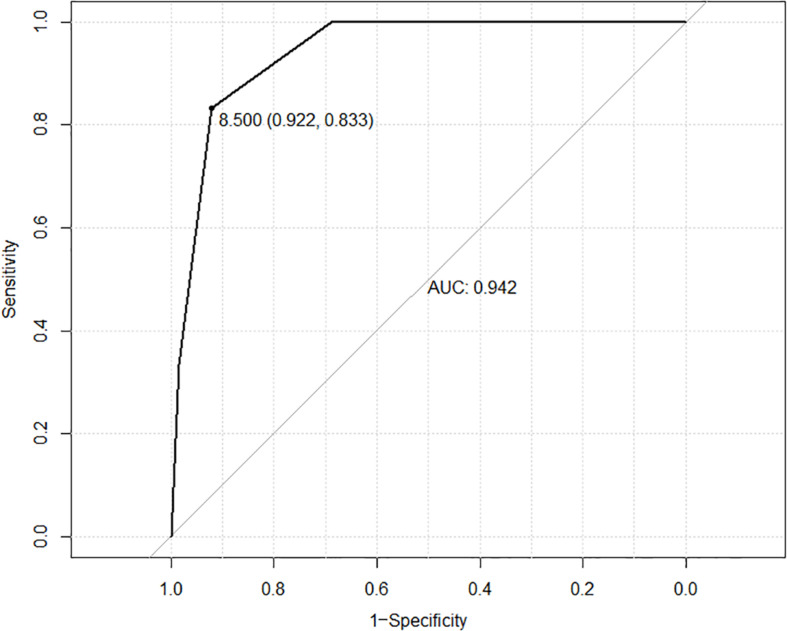
ROC curve based on RENAL score is shown in this figure. The AUC value was 0.942. The cutoff was 8. The 95% confidence interval was 0.833 to 0.922.

After univariate risk factor analysis, R.E.N.A.L. score (*P* < 0.001), diameter of tumor (*P* = 0.093), and ablation zone (*P* = 0.123), postoperative urine routine (RBC, *P* = 0.001; WBC, *P* = 0.255; and urine protein, *P* = 0.035), the distance between the ureter and tumor (*P* = 0.225), or ablation zone (*P* = 0.089), and total energy (*P* = 0.739) were included in the multivariate analysis of risk factors. After multivariate logistic regression analysis, only R.E.N.A.L. score (*P* < 0.001) was related to this complication. The ROC curve is shown in Figure 2. The AUC value was 0.942. The cutoff was 8. The 95% confidence interval was 0.833 to 0.922.

## Discussion

Stenosis of ureter after MWA for the treatment of renal tumor is a rare complication. Rarely had literature reported this complication. Chen et al. (17) had published a case report of ureteropelvic junction obliteration after RFA resulting in nephrectomy. Mansilla et al. (10) and Thompson et al. (12) reported one case and two cases ureteropelvic junction stricture after MWA, respectively. Schmitz et al. (18) have reported two cases of ureteropelvic junction stricture remote from the ablation site, which is similar to our study. All of these studies did not report the incidence rate and potential risk factors of this complication.

Our results showed that the R.E.N.A.L. score is associated with this complication. In contrast to previous research, which concluded the location of the tumor and growth pattern were independent predictors of developing stenosis of ureter (19). Our research did not find the location of tumor and growth pattern were related to it independently. Statistical analysis showed the diameter of the tumor is not associated with this complication. It is consistent with previous studies using MWA to treat renal mass (20–22). These studies did not report stenosis of ureter after MWA. This complication is irrelevant to ablation energy but relevant to the comprehensive information of location, and the distance between tumor and renal pelvis might indicate the reason of developing stenosis of the ureter is influenced not only by energy but also by the location of the lesion. It is possible for the heat to be transferred from the ablation zone to renal pelvis and heated the urine. Superheating urine injures the muscular layer and submucosa of the ureter and induces the stenosis of the ureter. Preoperative double-J stent placement may also help to avoid this complication after ablation, which might cause the superheating urine be carried away and the decrease in the rate of injury of the ureter. All of these patients did not experience the preoperative double-J stent placement, which might be the potential factor of this postoperative delayed complication. Hydrodissection was needed to protect the ureter. Previous research has proved that hydrodissection was an effective measure for protecting the tract adjacent renal mass (15). The combination of hydrodissection and preoperative double-J stent placement might be a better choice (23). Additionally, retrograde cold saline perfusion may also be a feasible method to decrease the temperature of urine and injury of the ureter (24). But there is no clear recommended flow rate. Moreover, placing the protective temperature-monitoring device into the renal pelvis to control the temperature of the urine could help the operator master the critical temperature precisely. Abnormal postoperative urine routine could indicate the ablation zone is communicated with the renal pelvis and inflammatory response because of injury of the ureter. Three of these patients were diagnosed with stenosis of the ureter more than 4 months after MWA therapy. There was no symptom of injury of the ureter after operation immediately, demonstrating that the stenosis of the ureter is delayed progress, which is corresponding to the previous study (25). One patient complained of abdominal pain 3 days after ablation because of ureterectasia of the upper ureter and achieved relief after accepted Double-J stent. Hence, postoperative double-J stent placement is also a remedial measure to alleviate the degree of stenosis of the ureter. It was also the treatment after injury of the ureter during surgical operation (26). Further study should focus on the protective measure of the ureter during ablation to minimize this complication, such as the combination of various protective methods.

There are still some limitations to this study—first, the nature of the retrospective study, which might affect the evaluation of outcomes. Second, because the stenosis of the ureter remote from the ablation site after MWA is a rare complication, the series of this complication is still limited; the power of evidence of risk factors is still low. Third, the experience of doctors and single-center study could impact the outcome.

In conclusion, R.E.N.A.L. score is associated with ureter stenosis after MWA for the treatment of renal tumor, which combines the information of location, depth, and size of tumor. Preoperative evaluation of the tumor is necessary for avoiding ureter stenosis. Moreover, the combination of various preoperative protective methods might be sufficient to reduce the rate of this complication such as preoperative double-J stent placement, hydrodissection technique, and retrograde cold saline perfusion. In addition, the postoperative remedy is also necessary to decrease the degree of stenosis of the ureter.

## Data Availability Statement

The datasets analyzed in this manuscript are not publicly available. Requests to access the datasets should be directed to wjpdabao@gmail.com.

## Ethics Statement

The studies involving human participants were reviewed and approved by Ethics Committee of PLA General Hospital. Written informed consent for participation was not required for this study in accordance with the national legislation and the institutional requirements.

## Author Contributions

JW: protocol and project development, methodology, data collection, manuscript writing, and manuscript editing. JY and ZC: protocol and project development, and methodology. ZH and XY: protocol and project development, and data management. KL and PH: data collection and management. FL and PL: protocol and project development, and manuscript review. All authors contributed to the article and approved the submitted version.

## Conflict of Interest

The authors declare that the research was conducted in the absence of any commercial or financial relationships that could be construed as a potential conflict of interest.

## References

[1] Capitanio U Bensalah K Bex A Boorjian SA Bray F Coleman J Epidemiology of renal cell carcinoma. *Eur Urol.* (2019) 75:74–84. 10.1016/j.eururo.2018.08.036 30243799 PMC8397918

[2] Capitanio U Montorsi F. Renal cancer. *Lancet.* (2016) 387:894–906. 10.1016/S0140-6736(15)00046-X 26318520

[3] Vander Eeckt K Joniau S Van Poppel H. Open surgery for localized RCC. *ScientificWorldJournal.* (2007) 7:742–52. 10.1100/tsw.2007.142 17619756 PMC5901146

[4] Zegel HG Holland GA Jennings SB Chong WK Cohen JK. Intraoperative ultrasonographically guided cryoablation of renal masses: initial experience. *J Ultrasound Med.* (1998) 17:571–6. 10.7863/jum.1998.17.9.571 9733175

[5] Motzer RJ Jonasch E Agarwal N Bhayani S Bro WP Chang SS Kidney cancer, version 2.2017, NCCN clinical practice guidelines in oncology. *J Natl Compr Canc Netw.* (2017) 15:804–34. 10.6004/jnccn.2017.0100 28596261

[6] Chen CN Liang P Yu J Yu XL Cheng ZG Han ZY Contrast-enhanced ultrasound-guided percutaneous microwave ablation of renal cell carcinoma that is inconspicuous on conventional ultrasound. *Int J Hyperthermia.* (2016) 32:607–13. 10.3109/02656736.2016.1172118 27269816

[7] Banerjee S Wang DS Kim HJ Sirlin CB Chan MG Korn RL A computed tomography radiogenomic biomarker predicts microvascular invasion and clinical outcomes in hepatocellular carcinoma. *Hepatology.* (2015) 62:792–800. 10.1002/hep.27877 25930992 PMC4654334

[8] Guan W Bai J Liu J Wang S Zhuang Q Ye Z Microwave ablation versus partial nephrectomy for small renal tumors: intermediate-term results. *J Surg Oncol.* (2012) 106:316–21. 10.1002/jso.23071 22488716

[9] Choi SH Kim JW Kim JH Kim KW. Efficacy and safety of microwave ablation for malignant renal tumors: an updated systematic review and meta-analysis of the literature since 2012. *Korean J Radiol.* (2018) 19:938–49. 10.3348/kjr.2018.19.5.938 30174484 PMC6082757

[10] Mansilla AV Bivins EE Jr. Contreras F Hernandez MA Kohler N Pepe JW. CT-guided microwave ablation of 45 renal tumors: analysis of procedure complexity utilizing a percutaneous renal ablation complexity scoring system. *J Vasc Interv Radiol.* (2017) 28:222–9. 10.1016/j.jvir.2016.10.013 27988263

[11] Castle SM Salas N Leveillee RJ. Initial experience using microwave ablation therapy for renal tumor treatment: 18-month follow-up. *Urology.* (2011) 77:792–7. 10.1016/j.urology.2010.12.028 21324512

[12] Thompson SM Schmitz JJ Thompson RH Weisbrod AJ Welch BT Viers BR Introduction of microwave ablation into a renal ablation practice: valuable lessons learned. *AJR Am J Roentgenol.* (2018) 211:1381–9. 10.2214/AJR.18.19775 30247980

[13] Kutikov A Uzzo RG. The R.E.N.A.L. nephrometry score: a comprehensive standardized system for quantitating renal tumor size, location and depth. *J Urol.* (2009) 182:844–53. 10.1016/j.juro.2009.05.035 19616235

[14] Gao Y Liang P Yu X Yu J Cheng Z Han Z Microwave treatment of renal cell carcinoma adjacent to renal sinus. *Eur J Radiol.* (2016) 85:2083–9. 10.1016/j.ejrad.2016.09.018 27776662

[15] Cheng Z Yu X Han Z Liu F Yu J Liang P. Ultrasound-guided hydrodissection for assisting percutaneous microwave ablation of renal cell carcinomas adjacent to intestinal tracts: a preliminary clinical study. *Int J Hyperthermia.* (2018) 34:315–20. 10.1080/02656736.2017.1338362 28641464

[16] Mauri G Nicosia L Varano GM Bonomo G Della Vigna P Monfardini L Tips and tricks for a safe and effective image-guided percutaneous renal tumour ablation. *Insights Imaging.* (2017) 8:357–63. 10.1007/s13244-017-0555-4 28500486 PMC5438321

[17] Chen SH Mouraviev V Raj GV Marguet CG Polascik TJ. Ureteropelvic junction obliteration resulting in nephrectomy after radiofrequency ablation of small renal cell carcinoma. *Urology.* (2007) 69:982.e3–5. 10.1016/j.urology.2007.02.031 17482951

[18] Schmitz JJ Schmit GD Viers BR Atwell TD. Renal microwave ablation resulting in ureteropelvic junction stricture remote from the ablation site. *J Vasc Interv Radiol.* (2017) 28:1278–80.e1. 10.1016/j.jvir.2017.03.010 28841931

[19] Wah TM Irving HC Gregory W Cartledge J Joyce AD Selby PJ. Radiofrequency ablation (RFA) of renal cell carcinoma (RCC): experience in 200 tumours. *BJU Int.* (2014) 113:416–28. 10.1111/bju.12349 24053769 PMC4233988

[20] Klapperich ME Abel EJ Ziemlewicz TJ Best S Lubner MG Nakada SY Effect of tumor complexity and technique on efficacy and complications after percutaneous microwave ablation of stage T1a renal cell carcinoma: a single-center, retrospective study1. *Radiology.* (2017) 284:272–80. 10.1148/radiol.2016160592 28076721 PMC5495130

[21] Bai J Hu Z Guan W Zhuang Q Wang S Liu J Initial experience with retroperitoneoscopic microwave ablation of clinical T(1a) renal tumors. *J Endourol.* (2010) 24:2017–22. 10.1089/end.2010.0204 20932080

[22] Schellhaas B Hammon M Strobel D Pfeifer L Kielisch C Goertz RS Interobserver and intermodality agreement of standardized algorithms for non-invasive diagnosis of hepatocellular carcinoma in high-risk patients: CEUS-LI-RADS versus MRI-LI-RADS. *Eur Radiol.* (2018) 28:4254–64. 10.1007/s00330-018-5379-1 29675659

[23] Garnon J Cazzato RL Caudrelier J Nouri-Neuville M Rao P Boatta E Adjunctive thermoprotection during percutaneous thermal ablation procedures: review of current techniques. *Cardiovasc Intervent Radiol.* (2019) 42:344–57. 10.1007/s00270-018-2089-7 30310986

[24] Margulis V Matsumoto ED Taylor G Shaffer S Kabbani W Cadeddu JA. Retrograde renal cooling during radio frequency ablation to protect from renal collecting system injury. *J Urol.* (2005) 174:350–2. 10.1097/01.ju.0000161596.71457.1b 15947688

[25] Selli C Turri FM Gabellieri C Manassero F De Maria M Mogorovich A. Delayed-onset ureteral lesions due to thermal energy: an emerging condition. *Arch Ital Urol Androl.* (2014) 86:152–3. 10.4081/aiua.2014.2.152 25017604

[26] Tavakoli A Surange RS Pearson RC Parrott NR Augustine T Riad HN. Impact of stents on urological complications and health care expenditure in renal transplant recipients: results of a prospective, randomized clinical trial. *J Urol.* (2007) 177:2260–4; discussion 2264. 10.1016/j.juro.2007.01.152 17509336

