# Supplementary material for: Risk Factors of Ureteral Stenosis After Percutaneous Microwave Ablation of Renal Tumor, a Single-Center Experience
Source: Front Oncol. 2020 Sep 18;10:521349. doi: 10.3389/fonc.2020.521349 (PMC7531279; doi:10.3389/fonc.2020.521349)
Supplement: Supplementary file 1 [file Table_1.docx]

**Supplementary Table 1.** Major and minor complication after MWA of renal tumor

| Complication | N = 211 | Complication |
| --- | --- | --- |
| I |  |  |
|  | 10(4.7%) | Fever |
|  | 23(10.9%) | Microscopic haematuria |
|  | 3(1.4%) | Gross hematuria |
|  | 14(6.6%) | Flank pain, and abdominal pain |
|  | 3(1.4%) | Perirenal bleeding |
|  | 3(1.4%) | Vomit |
|  | 1(0.5%) | Urinoma |
| II | 2(1%) | Diarrhea |
| IIIa |  |  |
|  | 3(1.4%) | pleural effusion or ascites |
| IIIb |  |  |
|  | 6(2.8%) | Thermal injury of pelvicalyceal system |
|  | 1(0.5%) | Colon perforation |
| IV/ V | 0 |  |
|  |  |  |
